# Circulating Levels of Soluble MICB in Infants with Symptomatic Primary Dengue Virus Infections

**DOI:** 10.1371/journal.pone.0098509

**Published:** 2014-05-28

**Authors:** Daniel H. Libraty, Lei Zhang, AnaMae Obcena, Job D. Brion, Rosario Z. Capeding

**Affiliations:** 1 Division of Infectious Diseases and Immunology, Department of Medicine, University of Massachusetts Medical School, Worcester, Massachusetts, United States of America; 2 Department of Medicine, Research Institute for Tropical Medicine, Manila, Philippines; 3 San Pablo City Health Office, San Pablo, Philippines; 4 Department of Microbiology, Research Institute for Tropical Medicine, Manila, Philippines; University of Rochester, United States of America

## Abstract

Dengue is the most prevalent arthropod-borne viral illness in humans. A MHC class I polypeptide-related sequence B (MICB) single nucleotide polymorphism (SNP) was previously associated with symptomatic dengue compared to non-dengue causes of acute febrile illnesses in infants. We measured circulating levels of soluble (s)MICB in the sera of infants with symptomatic primary dengue virus infections. We found that serum levels of sMICB increased between pre-infection and acute illness among infants with symptomatic primary dengue virus infections. The likelihood of being hospitalized with an acute primary DENV infection during infancy also tended to be higher with increasing acute illness sMICB levels. The elevation of sMICB during acute primary DENV infections in infants likely represents an immune evasion strategy and contributes to the severity of the acute illness.

## Introduction

Dengue is the most prevalent arthropod-borne viral illness in humans with half of the world's population at risk. The global burden of symptomatic dengue is on the order of 100 million cases/year [1]. The dengue viruses (DENVs) are single-stranded, positive-sense, RNA-containing enveloped viruses belonging to the *Flavivirus* genus within the Flaviviridae family [2]. There are four serotypes of DENVs (DENV1-4). DENV infections produce a wide spectrum of clinical illness. It ranges from asymptomatic or mild illness to a severe and potentially life threatening disease, dengue hemorrhagic fever (DHF)/dengue shock syndrome (DSS).

A genome-wide association study (GWAS) found that polymorphisms in only two genes were associated with the development of DSS compared to milder forms of symptomatic dengue in children and adults [3]. One of those genes was MHC class I polypeptide-related sequence B (MICB). The MICB single nucleotide polymorphism (SNP) rs3132468 was also associated with symptomatic dengue compared to non-dengue causes of acute febrile illnesses in children and adults, and in infants [4]. Proteolytic cleavage of MICB on cell surface membranes produces soluble (s)MICB that can be measured in sera [5]. We have been conducting a prospective clinical study of DENV infections during infancy in the Philippines [6]. We therefore examined circulating levels of sMICB in the sera of infants with symptomatic primary dengue virus infections. We found that serum levels of sMICB increased between pre-infection and acute illness among infants with symptomatic primary dengue virus infections. The likelihood of being hospitalized with an acute primary DENV infection during infancy also tended to be higher with increasing acute illness sMICB levels.

## Methods

### Ethics Statement

The study protocol was approved by the institutional review boards of the Research Institute for Tropical Medicine, Philippines, and the University of Massachusetts Medical School. Mothers and their healthy infants were recruited and enrolled after providing written informed consent.

### Clinical Study

The study began in January 2007 in San Pablo, Laguna, Philippines, and has been previously described [6]. Blood samples were collected from the infant and mother at the first study visit when the infant was between approximately 6-18 weeks old. Clinical and epidemiological information were collected at the study visit. We conducted surveillance year-round for hospitalized acute febrile illnesses in study infants across the seven hospitals serving San Pablo, Philippines. During the rainy season (June-November), mothers were encouraged to bring their infants to the San Pablo City Health Office for evaluation of outpatient febrile illnesses. Acute- and convalescent-phase (day 14) blood samples were obtained from study infants with febrile illnesses that did not have an obvious source at time of presentation (*e.g.* lobar pneumonia, bacterial meningitis, pyelonephritis).

A DENV infection was identified in febrile infants by serotype-specific RT-PCR in acute-phase sera and DENV IgM/IgG ELISA in paired acute and convalescent phase sera. Primary or secondary DENV infections were identified by previously established serologic criteria for the paired IgM/IgG ELISA results [7]. The infecting DENV serotype was identified by RT-PCR for all the symptomatic infants.

### sMICB ELISA

Briefly, capture Ab to human MICB (R&D Systems, 20 µg/ml) was coated on 96-well flat-bottomed microtiter plates (Thermo Scientific). Plates were blocked with bovine serum albumin (BSA). Undiluted sera (50 µl/well) were added to the plates and incubated for 2 h at room temperature. Then, biotin-conjugated detecting Ab to human MICB (R&D Systems, 2 µg/ml) was added followed by streptavidin-horseradish peroxidase (HRP). The ELISA plates were developed using SuperSignal ELISA Femto Substrate (Pierce Protein Biology). sMICB levels were determined using a luminometer (Envision 2012 plate reader, Perkin Elmer).

### Statistical Analysis

The SPSS software package (version 21.0) was used for statistical analyses. Comparisons between pre-infection and acute illness paired sMICB levels were made using the Wilcoxon signed rank non-parametric statistical test. P<0.05 was considered significant; 0.05≤p<0.10 was considered a significant trend.

## Results

### Symptomatic primary DENV infections in infants

The characteristics of the infants with symptomatic primary DENV infections are shown in Table 1.

**Table 1:** Characteristics of infants with symptomatic primary dengue virus (DENV) infections.

Number of infants	*n* = 46
Age at time of DENV infection (median [95% confidence interval])	7.4 [5.8–8.6] months
Gender (male:female)	23∶23
Serotype of dengue virus infection	DENV1: *n* = 5 DENV2: *n* = 6 DENV3: *n* = 33 DENV4: *n* = 2
Disease severity	Hospitalized: *n* = 24 Outpatient: *n* = 22

### Circulating levels of sMICB in infants with symptomatic primary DENV infections

**Figure 1:**
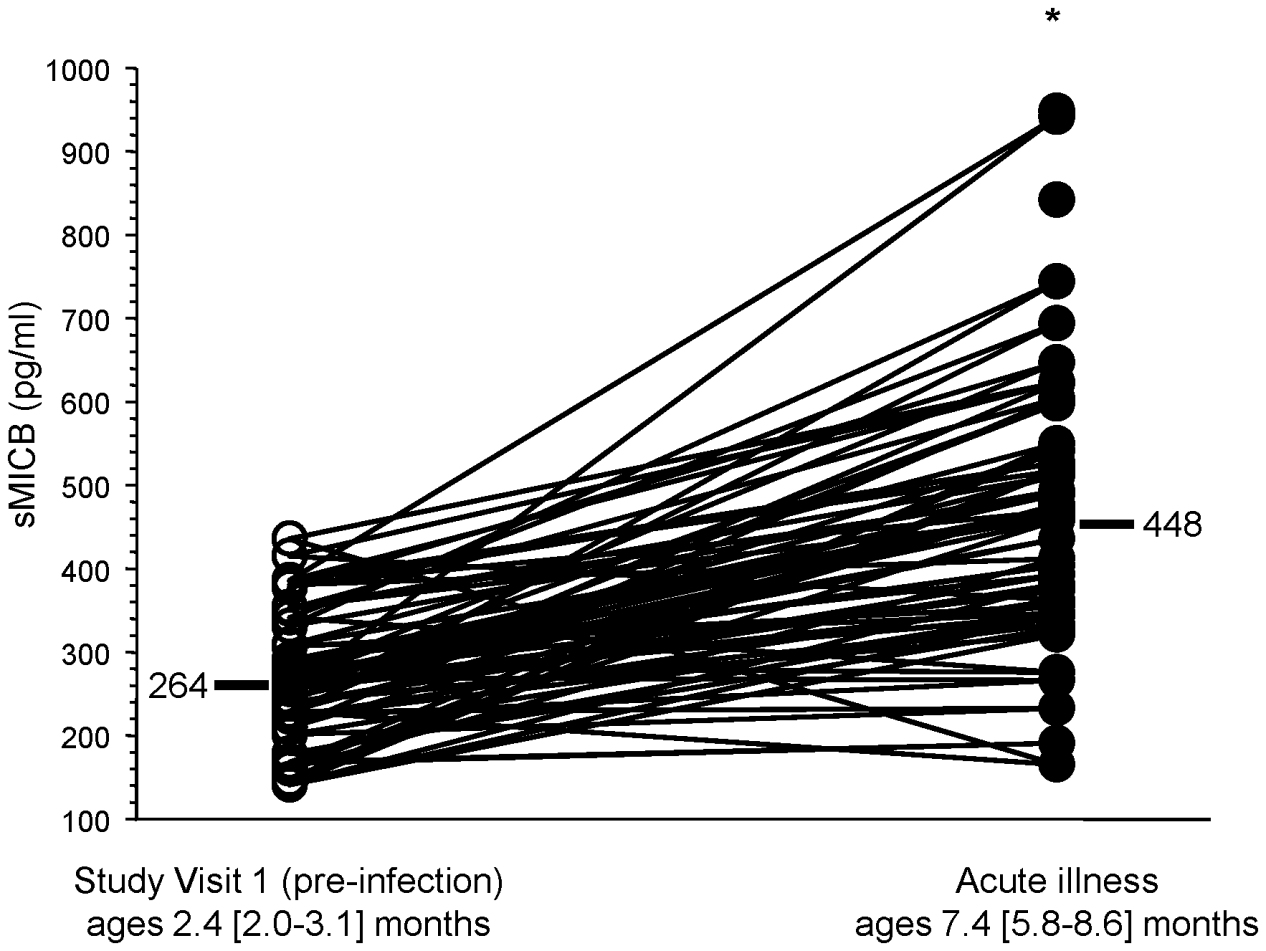
Serum levels of soluble (s)MICB pre-infection and during acute illness among infants with symptomatic primary dengue virus infections (*n* = 46). Ages are shown as median [95% confidence interval]. Bars are median values. * p<0.001 for comparison between the two groups.

**Figure 2:**
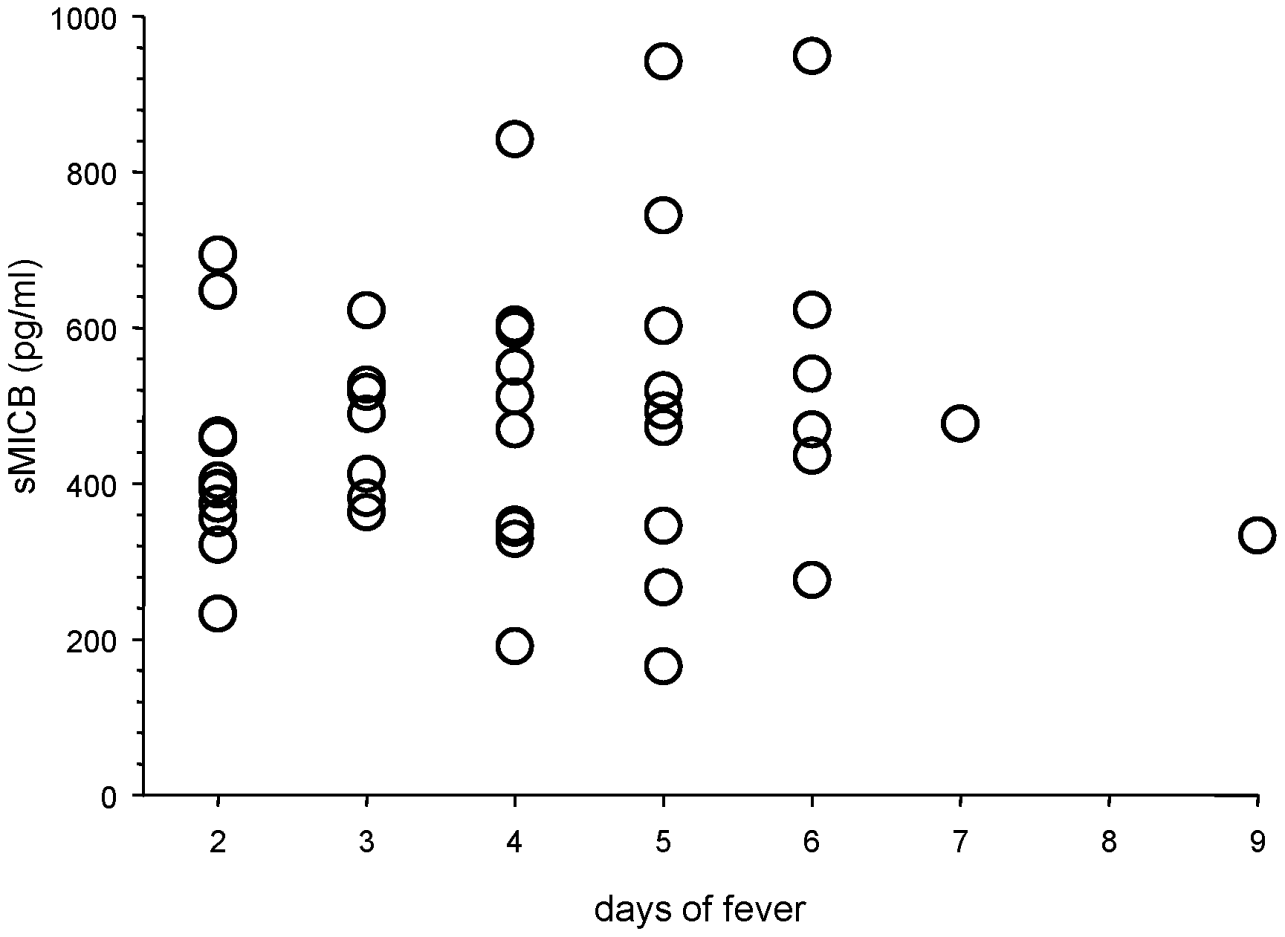
Serum levels of soluble (s)MICB during acute illness by day of fever among infants with symptomatic primary dengue virus infections (*n* = 46).

Circulating levels of sMICB increased between the pre-infection levels at study visit 1 and acute illness levels among infants with symptomatic primary DENV infections (Figure 1). sMICB levels were not different over the first week of illness among infants with symptomatic primary DENV infections (Figure 2).

### sMICB levels trend higher in infants hospitalized with primary DENV infections

In a binary logistic regression model, the odds of a symptomatic primary DENV infection during infancy that led to hospitalization trended higher for every 100 pg/ml increase in acute illness serum sMICB (odds ratio (OR) [95% confidence interval] for hospitalization compared to outpatient illness: 1.4 [0.97–2.1], p = 0.075, *n* = 46).

## Discussion

MICB is a cell surface protein and a ligand for the natural killer group 2 member D (NKG2D) receptor, a stimulatory receptor on NK cells and a co-stimulatory receptor on CD8+ T-cells [8]. sMICB blocks the activating signal produced by MICB-NKG2D, and is felt to be an immune evasion strategy [5]. We found that circulating levels of sMICB increased during acute primary symptomatic DENV infections in infants compared to pre-infection levels. The elevation of sMICB during acute DENV infections is likely an immune evasion strategy in a systemic viral infection. It could inhibit activation of the anti-viral effects of NK cells, CD8+ T-cells, or both, in infants with primary DENV infections and thereby contribute to the development of an acute febrile illness. We also noted that the circulating levels of sMICB in healthy 2 month old infants were higher than what has been reported in healthy adults [9], [10]. This may contribute to the susceptibility of infants to severe dengue with primary infections.

The likelihood of being hospitalized with an acute primary DENV infection during infancy tended to be higher with increasing acute illness sMICB levels. The MICB polymorphism (MICB SNP rs3132468) associated with symptomatic dengue in infants is an A/G change in an intron region of the human MICB gene (dbSNP database, NCBI). The effect of this SNP is unknown, but we postulate it could promote the proteolytic cleavage of cell surface MICB leading to increased sMICB levels. Further experiments to examine the effect of this SNP are planned.
